# The Synthesis and Characterization of Novel Bi-/Trimetallic Nanoparticles and Their Nanocomposite Membranes for Envisaged Water Treatment

**DOI:** 10.3390/membranes10090232

**Published:** 2020-09-14

**Authors:** Lwazi Ndlwana, Keneiloe Sikhwivhilu, Richard Motlhaletsi Moutloali, Jane Catherine Ngila

**Affiliations:** 1 Department of Chemical Sciences, Faculty of Science, University of Johannesburg, P. O. Box 17011, Doornfontein, Johannesburg 2028, South Africa; ndlwana@gmail.com (L.N.); rmoutloali@uj.ac.za (R.M.M.); jcngila2015@gmail.com (J.C.N.); 2 DSI/Mintek Nanotechnology Innovation Centre, Advanced Materials Division, Mintek, 200 Malibongwe Drive, Randburg, Johannesburg 2125, South Africa

**Keywords:** catalytic nanocomposite membranes, hyperbranched polyethyleneimine, polyethersulfone membranes, stabilized nanoparticles

## Abstract

The impact of worldwide water scarcity, further exacerbated by environmental pollution, necessitates the development of effective water treatment membranes. Herein, we report the synthesis and characterization of nanocomposite membranes containing hyperbranched polyethyleneimine (HPEI) stabilized bi-and trimetallic nanoparticles. These membranes were prepared by blending a pre-grafted Polyethersulfone (PES) powder with the Pd@Fe@HPEI and Pd@FeAg@HPEI nanoparticles followed by phase inversion. The membranes, together with stabilized nanoparticles, were characterized by several analytical techniques, such as attenuated total reflectance–Fourier transform infra-red spectroscopy (ATR-FTIR), X-ray photoelectron spectroscopy (XPS), X-ray diffractometry (XRD), optical contact angle (OCA), scanning electron microscopy (SEM), atomic force microscopy (AFM), and high-resolution transmission electron microscopy (HRTEM). These techniques revealed the elemental composition, zerovalent nature of the nanoparticles, and their small and even size distribution. Surface analysis showed chemical bonding between the polymeric functional groups and the supported nanoparticles. Furthermore, the nanocomposite membranes were found to be hydrophilic. Additionally, the membranes were investigated for swelling (water uptake), porosity, pore size, pure water permeation fluxes, and they indicated a decreased protein adhesion property. As such, the membranes fabricated in this work indicate the required properties for application in water treatment.

## 1. Introduction

Persistent organic compounds in water, such as dyes and chlorinated organics, are highly toxic with severe endocrine disrupting effects in biota [1]. Nanozero-valent metals (nZVMs) have been found to be critical in the elimination of these environmental insults. These nZVMs are widely used for the removal of unwanted materials given their ability to not only degrade these compounds, but also their by-products [2]. The demerit of these nanoparticles is in their agglomeration when unsupported, and in leaching which creates secondary pollution. Consequently, these nanoparticles need to be supported with suitable materials such as polyligands, and this is best done in polymeric membranes like polyethersulfone (PES), polysulfone (PS), and polyvinylidenedifluoride (PVDF) [3]. Dendritic polymers such as hyperbranched polymers of polypropyleneimine (HPPI) and polyethylene imine (HPEI) in membranes have become synonymous with the chelation and removal of metals from water. Subsequently, this ability has been exploited in the encapsulation/stabilization of metal nanoparticles; and when supported on membranes, these are powerful in the removal of toxic organics. Malinga (2013) reported on a blended conjugated system of PPI/cyclodextrin and bimetallic Fe/Ni nanoparticles into a polysulfone (PS) membrane matrix with the ability to remove 2,4,6-trichlorophenol in water [4]. Bimetallic nanocomposite membrane systems in the degradation of POPs have been reported before, and the subsequent production of by-products with noted toxicity observed [5]. The use of membrane-based AOP approaches such as Fenton and Fenton-like reactions are favored for the latter because of affordability, and reagents (Fe and H_2_O_2_) are readily available. In this regard, bi- and trimetallic nanocomposite membrane systems may result in more complete reactions via a multicatalytic approach in simultaneous reductive/oxidative degradation and mineralization of pollutants [6].

The aim of this work was to develop a new synthesis method for polymethacrylic acid (PMAA)-PES nanocomposite membranes containing HPEI-stabilized Pd@Fe and Pd@FeAg nanoparticles, for enhanced membrane properties. The new nanocomposite membranes were be prepared and characterized similarly to nanoparticles and membrane materials envisaged for water treatment [5,6]. Among the membrane properties investigated are morphology, hydrophilicity, surface tension, surface free energy, water uptake, porosity, pore size, and protein adhesion.

## 2. Materials and Methods

### 2.1. Materials

PES 3100P (31–58 kDa) was obtained from Solvay (Beveren, Belgium). Polyvinylpyrrolidone 40K (PVP, 98%), N-methyl-2-pyrrolidone, hyperbranched polyethyleneimine (HPEI, 25K (99.8%), iron sulfate heptahydrate (FeSO_4_•7H_2_O, 98%), isopropanol (IPA, ACS reagent), sodium borohydride (NaBH_4_, 98%), silver nitrate (AgNO_3_, 99.0%), palladium acetate (Pd(OAc)2, 99.8%), sodium hydroxide (NaOH pellets, 99.8%), hydrogen peroxide (H_2_O_2_, 30%), hydrochloric acid (HCl, 37%), and ethanol (GC grade, 99.9%) were all purchased from Sigma-Aldrich (Kempton Park, South Africa), and used without further purification. All deionized water (18.2 MΩcm resistivity) used was supplied by a Millipore^®^ (Tokyo, Japan) water system.

### 2.2. Preparation of the Nanoparticles and Nanocomposite Membranes

#### 2.2.1. One-Pot Synthesis of HPEI-Stabilized Zerovalent Nanoparticles

A borohydride reduction method as previously reported in other works [7,8] was modified for the synthesis of HPEI-stabilized Fe@Pd and Pd@FeAg nanoparticles.

A 1.0% (m/m) HPEI solution was prepared in a 100 mL solution of equal amounts of methanol and water and stirred for 15 min under N_2_ gas, 400 rpm at 25 °C. Subsequently, 50 mL of 2.0 M FeSO_4_•7H_2_O was added and stirred for 30 min to chelate the Fe^2+^. This resulted in the formation of a greenish color, following which a 50 mL solution of 3.2M NaBH_4_ was added over a period of 5 min, resulting in a black colloidal solution. The reaction then continued for a further 45 min. The Pd salt was weighed at 1.0% (m/m) FeSO_4_•7H_2_O and dissolved in 50 mL ethanol/ water (9:1) solution and added to the colloidal solution and allowed to react for 15 min. The nanoparticles yielded were magnetic and hence were collected using a magnetic stirrer bar and the supernatant discarded. This was followed by two 5-min centrifugation cycles in ethanol to remove excess HPEI, borohydride, and other unwanted by-products. These were then dried at 60 °C for 24 h and stored in a UV-proof desiccator cabinet (Scienceware ® Secador ®, Sigma-Aldrich, Kempton Park, South Africa).

The same procedure was followed for the preparation of HPEI-stabilized Pd@FeAg nanoparticles with the exception that 0.556 g of Ag precursor (10% m/m of the Fe precursor) was introduced and the two salts introduced simultaneously to the HPEI solution. Additionally, as Ag is photosensitive, a UV-proof reaction vessel was used to mitigate photodegradation. Post-coating with Pd also followed the same procedure as above and the same with washing, drying and storage.

#### 2.2.2. Nanocomposite Membrane Fabrication

The PMAA-PES powder was prepared using a microwave-assisted synthesis method, as developed by our research group [9]. The grafted powder was synthesized at ca. 16% degree of grafting and 50% crosslinking. The PMAA-PES was dissolved in NMP and then blended with the HPEI-stabilized nanoparticles (500 mg) by stirring and heating at 60 °C for 2 h for uniform dispersion in the solution. The resulting solutions had a composition of 16% (m/m) ofPMAA-PES, 0.5% (m/m) PVP, with predetermined nanoparticlesloadings. The solutions, now black in color with the dispersed nanoparticles, were cooled to room temperature and degassed under electric vacuum for 2 h to remove trapped air. The solutions were drawn down on a clean glass plate using a casting knife set at an air gap of 250 µm. The glass plate was then immersed in a coagulation bath of a water/IPA solution (4:1 *v*/*v*) for 24 h. This composition of the coagulation bath served to minimize not only the oxidation of the incorporated nanoparticles, but also the formation of macrovoids during rapid liquid–liquid exchange which would otherwise occur in water alone. The newly prepared membranes were then labeled according to their modification as presented in Table 1:

### 2.3. Characterization of the Composite Membranes

#### 2.3.1. ATR-FT-IR Analysis

Surface functionality of the nanoparticles and membranes was investigated by ATR-FTIR (Perkin Elmer Spectrum 100, PerkinElmer Inc., Waltham, MA, USA) set between 650 and 3700 cm^−1^ over 16 scans at a resolution of 4 cm^−1^. The membranes were dried in the desiccator for 24 h prior to all analyses.

#### 2.3.2. XPS Analysis

To corroborate the ATR-FTIR data, X-ray photoelectron spectroscopy using the Phoibos 150 electron energy analyzer (XPS, SPECS GmBH, Berlin, Germany) with a monochromatisized Al Kα photon energy source (1486.71 eV), was used for all nanoparticle compositional analyses. Survey scanning required an overall energy resolution of 0.8 eV, while individual core level spectra required 0.6 eV. Surface charging was counteracted by low-energy flood gun irradiation at 2.8 eV at an emission current of 20 µA during examination.

#### 2.3.3. XRD Analysis

The nanoparticles and membranes were investigated for their crystalline or amorphous nature using a Rigaku Ultima IV X-ray diffractometer (Rigaku Corp., Tokyo, Japan) equipped with a copper radiation source (K-α, 40 kV using a current of 40 mA). The samples were fitted onto quartz holders positioned on a spinning stage. The spectra were produced at a step of 0.01° and scan rate of 1.0°/min.

#### 2.3.4. ICP-OES Analysis

The metal content of the nanocomposite membranes was determined using ICP-OES (iCAP Duo 6500, Thermo Fisher Scientific, Waltham, MA, USA). Each membrane piece (5.05 cm in diameter) was cut into small portions and digested over 24 h at room temperature in 10 mL aqueous solutions of 25% *v*/*v* HNO_3_ prior to analysis.

#### 2.3.5. SEM Analysis

SEM (Tescan Vega 3 LMH SEM, Tescan Brno s.r.o, Brno, Czech Republic) was used to investigate the surface morphology due to the modification. For surface analyses, the membranes showing the filtration layer (surface) were carbon-coated using the Q150T ES carbon coater (Quorum Technologies, Lewes, UK) and subsequently analyzed. For cross-sectional analysis, the membranes were cut to 0.5 cm × 2.0 cm strips, and dipped in liquid nitrogen for about 3 min. These were subsequently broken apart to reveal the microporous structure. This was followed by carbon-coating before analysis.

#### 2.3.6. AFM Analysis

Topological analysis was carried out using AFM (Nanoscope IV microscope controller, Veeco Metrology Group, New York City, NY, USA), where the non-contact mode was used. Membrane surface roughness was obtained by analyzing 1 cm × 1 cm square pieces on several chosen spots).

#### 2.3.7. HRTEM Analysis

The nanoparticle size and size distribution were determined by use of HRTEM (JEM2100, Jeol, Tokyo, Japan). Firstly, the nanoparticles were sonicated in ethanol spotted on carbon-coated copper grids, and then mounted onto the instrument for analysis. The electron beam was set at 200 KV for all analyses. Image J software (v1.45, National Institute of Health, NIH, Bethesda, MD, USA) was used to measure the average size and size distribution of the nanoparticles from the data obtained from the analysis.

#### 2.3.8. Membrane Hydrophilicity, Surface Tension, and Surface Free Energy Analyses

Membrane hydrophilicity analysis was carried out on wet membranes using the camera-assisted goniometer (OCA20, Dataphysics Instruments, Charlotte, NC, USA) where water sessile drops (5 µL) were dispensed from a 200 µL syringe. The results were averaged from six different points on the surface of each membrane. Three pieces of each membrane were tested to minimize errors. Surface energy (Δ*G*) is another important parameter for investigating the wettability and extent of water-membrane interactions. These measurements were also taken using this same instrument, applying the van Oss acid–base theory. The liquids used were deionized water (polar solvent) and diiodomethane (dispersant). Drops of these solvents were then dispensed onto the membranes to determine Δ*G*.

#### 2.3.9. Water Uptake, Pore Size, Porosity, and Pure Water Permeation Fluxes

The membranes were investigated for water uptake to determine swelling and pure water permeation fluxes. The methodology followed to determine these properties has been reported in our previous work [9]. To determine water uptake and porosity, the fluid saturation method (gravimetric) was used to determine water adsorption. For pure water permeation flux measurements, the membranes were cut into 5.05 cm diameter pieces and placed into a stirred dead-end cell system (Sterlitech Corp., Kent, WA, USA). The membranes were compacted at 200 kPa and fluxes measured at 100 kPa. Pore size was also determined gravimetrically, using all the data obtained above in the Guerout–Elford–Ferry formula as shown in Equation (1) below:

(1) $$r_{m}={(\frac{\left(2.9-1.75\varepsilon \right)\times 8\eta lQ}{\varepsilon A\Delta P}\;)}^{1/2}$$

where η is water viscosity at 8.9 × 10^−4^ Pa s, and *P* is the operating pressure at 100 kPa. Porosity is represented by the symbol (ε), and Q is the permeate volume. The dimension l is the membrane thickness as measured on the ImageJ software (v1.4.2, NIH.gov), and A is the effective membrane area in m^2^.

#### 2.3.10. Protein Adhesion

Protein adhesion is another critical property, as it provides predictions of membrane–foulant interactions. One of the most used model foulants is bovine serum albumin (BSA), which possesses pronounced interactions with hydrophobic (or relatively hydrophobic) surfaces of sulfone polymers like PES [4]. These can lead to the build-up of unwanted materials (fouling) such as biofilms on the membrane pores and surfaces [9]. As such, BSA adsorption studies were carried out gravimetrically to measure the effect of the modification of the membrane on its protein adhesion character. The membranes were cut into 3 cm × 3 cm pieces and inserted into 1000 ppm solutions of BSA (adjusted to pH 7) by batch-mode shaking for 24 h. The membranes were then removed, rinsed, dried, and then weighed.

## 3. Results and Discussion

### 3.1. Surface Chemistry

**Figure 1:**
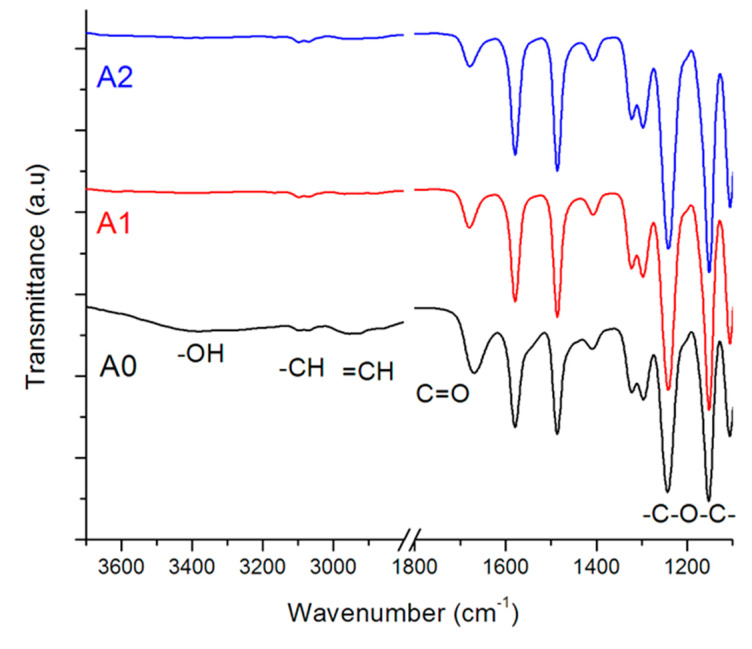
ATR-FTIR spectra of the membranes: PMAA-PES (A0), Pd@Fe@HPEI/PMAA-PES (A1), and Pd@FeAg@HPEI/PMAA-PES (A2).

Surface functionality was investigated on ATR-FTIR as shown on Figure 1. Characteristic functional groups of PES were observed at 1100–1240 for phenylene ethers, and 1300–1320, 1479 and 1586 cm^−1^ on all the membranes and these were ascribed to aromatic CH– where the PMAA side chains are chemically bonded. All these bands intensified with the presence of the HPEI-stabilized nanoparticles due to interactions between the latter and PMAA-PES. Membrane A0, the starting material, exhibited carbonyl bands from the carboxylic MAA groups at around 1670 cm^−1^ The inclusion of nanoparticles resulted in narrow shifts in the carbonyl band to higher wavenumbers due to the strong electrostatic interactions between the carboxyl groups and nanoparticles (from 1672–1684 cm^−1^), and this shift also indicated conjugation of carbonyl groups in PMAA-PES. A great reduction in the intensity of the CH– bands from 2875–3103 cm^−1^ was also observed, and this was attributed to steric strain as introduced by the nanoparticles on the PMAA-PES backbone. The disappearance of the carboxylic hydroxyl bands at 3390 cm^−1^ further confirmed electrostatic interactions between the nanoparticles and the carboxylic groups on PES [9,10]. The introduction of HPEI through the nanoparticles did not change the membrane surface functionality as no new bands appeared. This was anticipated given the low amounts of HPEI present after the washing of the nanoparticles, and the lack of covalent bonding between HPEI and PMAA-PES. This inconspicuous nature of HPEI in blended sulfone membranes has been reported previously [11].

**Figure 2:**
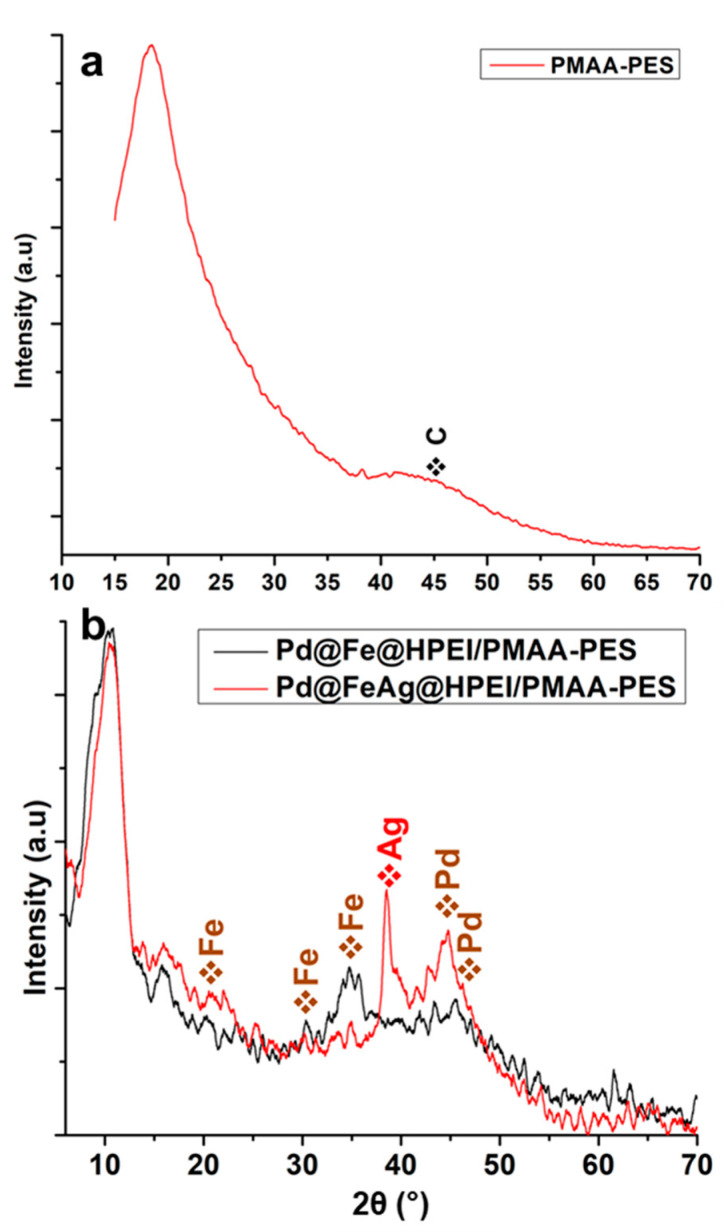
XRD spectra of the (**a**) PMAA-PES powder, and (**b**) the nanocomposites Pd@Fe@HPEI/PMAA-PES (black) and Pd@FeAg@HPEI/PMAA-PES (red). The Fe and Pd nanoparticles are labeled in brown since these are present in both nanocomposites. Ag is labeled in red for Pd@FeAg@HPEI/PMAA-PES.

The crystalline phases of the nanoparticles and membranes are presented on the XRD spectra in Figure 2. In Figure 2a, the PMAA-PES composite, as anticipated, exhibited a largely amorphous character. The chemically bound PMAA, like other acrylic moieties, is also amorphous in nature and hence the broad graphitic carbon peak observed at 45° [12]. Figure 2b shows that after the inclusion of the nanoparticles, the carbon containing HPEI was detected at 2Ɵ = 10° as it is also an amorphous material. Peaks at 2Ɵ = 20.0 (400), and 30.0° (440) were observed and these indicated the presence of Fe which contained the oxidized phase (Fe_3_O_4_) within the Pd@Fe@HPEI/PMAA-PES membrane matrix. The zerovalent phase was also observed at Bragg angles at ca. 35.2° for Fe (200), (111) and (211) at 42°. Reflections were observed at 44.8° and attributed to zerovalent Pd [13]. The Pd (002) at 2Ɵ = 45.5° has also been previously reported as a combinatorial phase between Fe and Pd in the synthesis of bimetallic nanoparticles of such elemental compositions. These Fe/Pd peaks lacked long-range order and suffered broadening due to the amorphous nature of PMAA-PES and the nanoparticles [12,14,15,16,17]. These peaks also appear in Pd@FeAg@HPEI/PMAA-PES, with enhanced crystallinity, and thus minor shifts. These shifts can be attributed to the presence of Ag, where the peaks appear to be narrower and more defined. At 2Ɵ = 38.5°, Ag (222) reflections appear, and according to the literature, FeAg nanoparticles present a similar profile [18,19]. The palladization of the FeAg nanoparticles resulted in the appearance of Pd reflections, like the previously discussed Pd@Fe@HPEI/PMAA-PES. Similar observations have been reported for the palladization of Fe and Ag-based nanoparticles in the literature [18,19,20].

**Figure 3:**
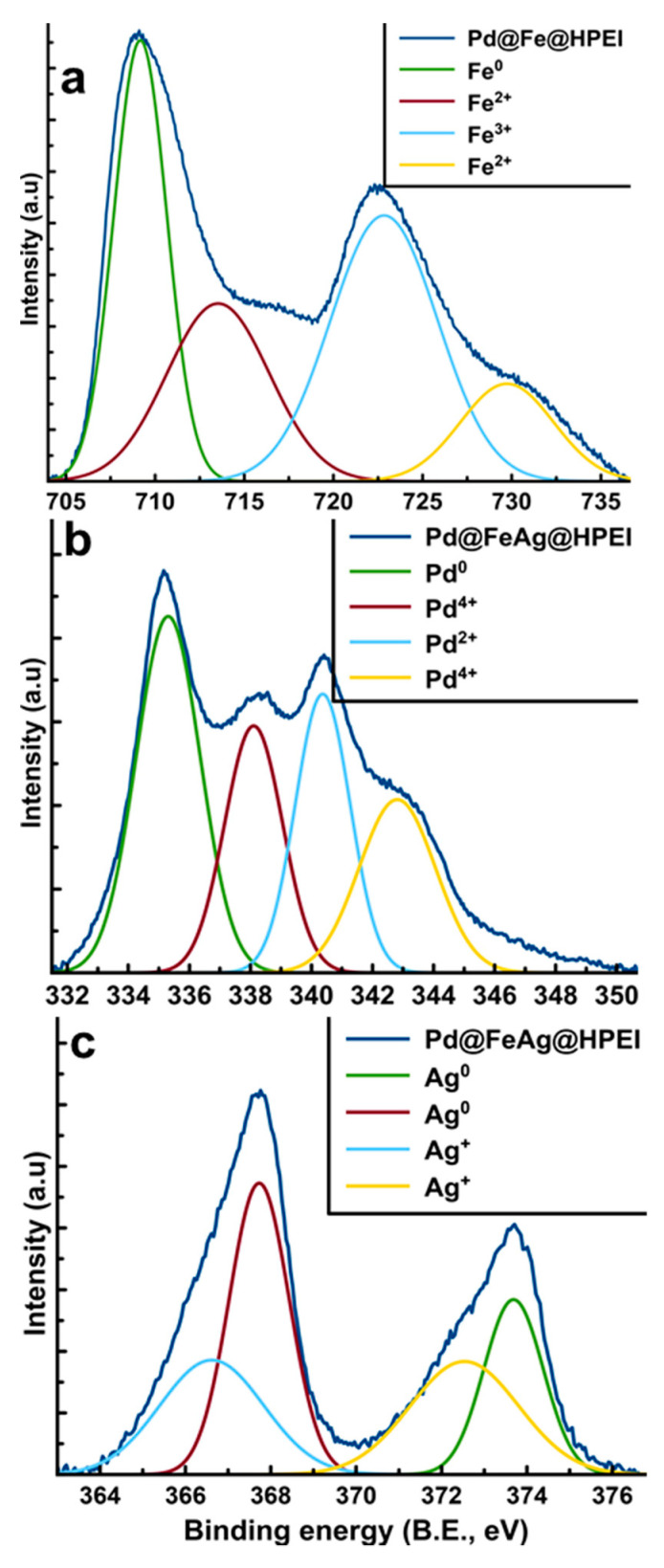
Deconvoluted XPS spectra of the prepared HPEI-stabilized nanoparticles: (**a**) Fe species Pd@Fe@HPEI; (**b**) Pd in Pd@FeAg@HPEI; and (**c**) Ag in Pd@FeAg@HPEI.

Figure 3 illustrates the XPS spectra of the nanoparticles used in the fabrication of the nanocomposite membranes presented in this study. Figure 3a depicts the peaks for the Fe species where oxidation was evident, with Fe^3+^ appearing at 722.8 eV with a satellite at 729.7 eV. This revealed the existence of two oxidation states, indicating that zerovalent iron was prevalent at 709.2 eV and the oxidized species (Fe^2+^) appearing at 713.5 eV. This means that oxidation of Fe^0^ may have occurred during synthesis, hence the presence of Fe^2+^. The appearance of Fe^2+^ has also been credited to the inclusion of other metals (Pd and later, Ag), and this results in lattice distortions to Fe [19]. Figure 3b presents the Pd species from Pd@FeAg@HPEI appearing at 335.3, 338.1, 340.4, and 342.8 eV. The first peak indicates the existence of metallic palladium (Pd^0^), with the rest of the peaks confirming the presence of ionic Pd [20,21]. Figure 3c presents the Ag species in Pd@FeAg@HPEI; the peaks at 366.6 and 372.5 correspond to ionic silver. The peaks at 367.4 and 373.3 were ascribed to the presence of metallic silver (Ag^0^) [22,23]. These findings are in line with literature data for the preparation of Fe-based zerovalent nanoparticles [24].

### 3.2. Membrane and Nanoparticle Morphology

**Figure 4:**
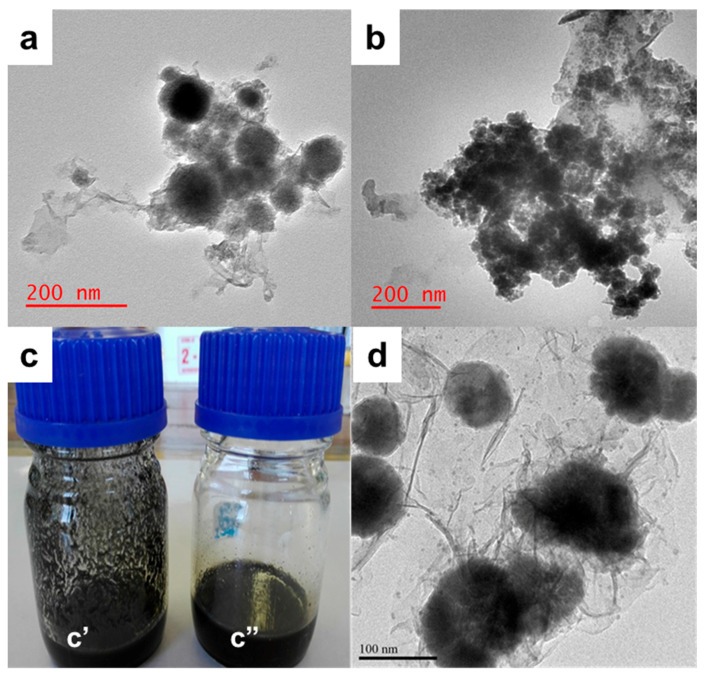
HR-TEM of (**a**) Pd@Fe@HPEI and (**b**) Pd@FeAg@HPEI, (**c**) The effect of HPEI on the dispersion of nanoparticles, where (**c’**) and (**c”**) are the respective Pd@Fe@PMAA-PES and Pd@Fe@HPEI/PMAA-PES casting solutions. (**d**) Pd@Fe@PMAA nanoparticles.

With the aim of obtaining the desired final nanocomposite membrane properties, the physical properties of the nanoparticles need to be understood. Additionally, chemical interactions between the stabilized nanoparticles and the membrane matrix at the molecular level is important to arrive at the required properties. Figure 4 illustrates the morphology, size, and shape of the Pd@Fe and Pd@FeAg nanoparticles as affected by the macromolecular stabilizer prior to membrane fabrication. The histogram of the size distribution is presented as Supplementary Information (Figure S1). Figure 4a presents the Pd@Fe@HPEI nanoparticles, and as can be observed, these possessed a largely spherical shape and an average size of 57 ± 9 nm with a size distribution of 29–114 nm. Figure 4b illustrates similarly shaped Pd@FeAg@HPEI nanoparticles, and these presented an average size of 28 ± 3 nm (16–51 nm), which are of smaller size as compared to Pd@Fe@HPEI. This observation can be attributed to improved chelation, as discussed in the previous subsections, for the XPS and enhanced crystallinity in the XRD data [5,10,12,16,18,19,20,21,22,25]. The stabilizer, HPEI proved to possess enhanced nanoparticle stabilization properties as 57 nm and 28 nm sized bi- and trimetallic nanoparticles were successfully prepared in this study. These were observed to be much smaller than the reported sodium carboxymethyl cellulose stabilization method for Fe-Pd nanoparticle preparation, where sizes of 85–97 nm were reported [26]. Figure 4c presents the unstabilized Pd@Fe@PMAA-PES casting solution (Figure 4c’) contrasted against that of the Pd@Fe@HPEI/PMAA-PES (Figure 4c”) to show the differences in nanoparticle dispersion in the PES-PMAA casting solutions. As presented, the former casting solution shows visible nanoparticle agglomerations as compared to the latter. This can be attributed to the stabilization capability of HPEI during the preparation step as opposed to PMAA-based macromolecular stabilization. Figure 4d presents the Pd@Fe@PMAA nanoparticles which possessed a much larger size of 93 ± 15 nm (47–157 nm) as compared to those stabilized with HPEI with a diameter of 28 ± 3 nm. This observation is attributed to chemical interactions between the carboxylic groups and the amine functionalities on HPEI in the casting solution blends. Furthermore, XPS confirmed the metal-carboxylic functionality bonding, which further aids stabilization within intermolecular make-up of the casting solutions. For these reasons, the preparation method as presented in this work is critical towards highly reactive nanocomposite membranes in the degradation of POPs in comparison to existing methods [5,17].

**Figure 5:**
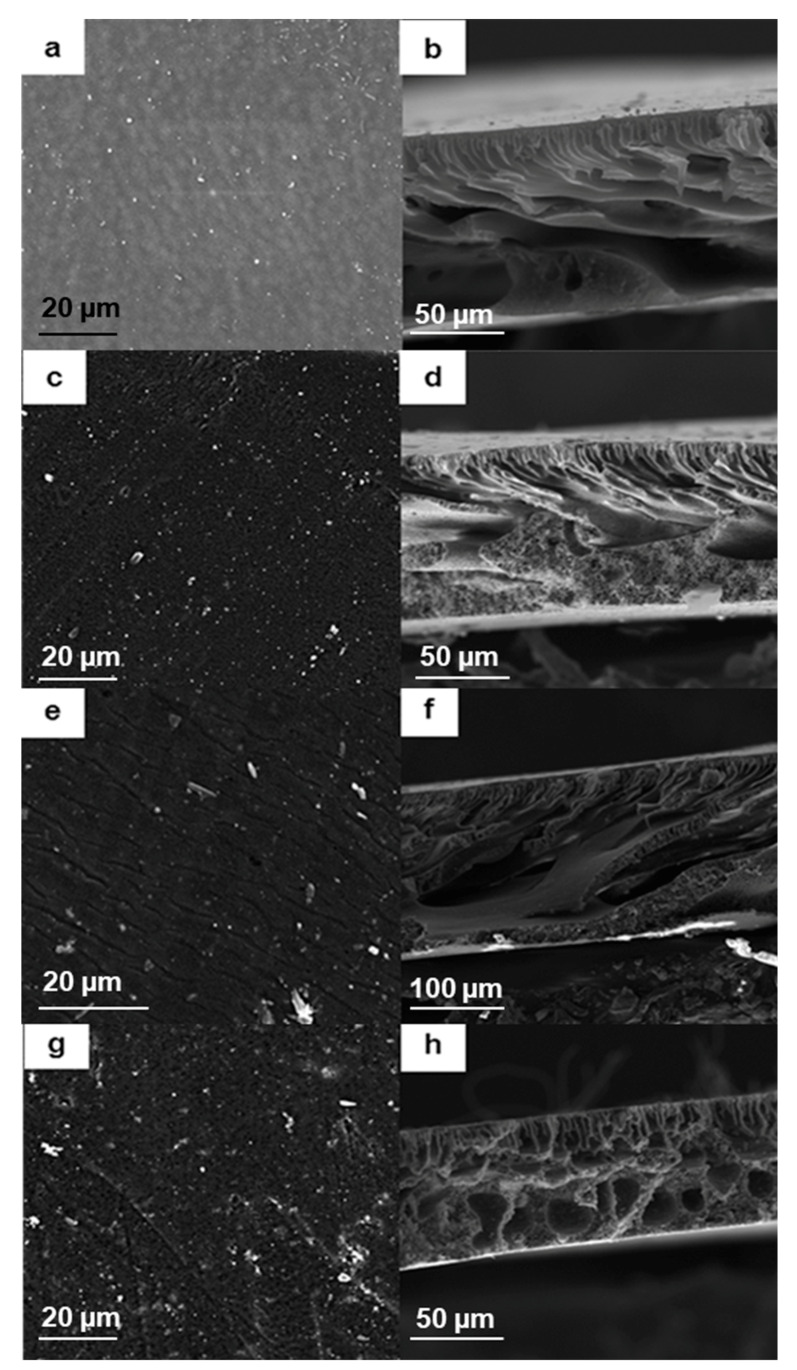
SEM images showing the respective surface and cross-sectional morphology of the membranes: (**a**,**b**) Pure PES; (**c**,**d**) A0; (**e**,**f**) A1; and (**g**,**h**) A2.

The membranes were also characterized with SEM for surface and cross-sectional morphology as presented in Figure 5. The pristine membrane, prepared from pure PES (shown in Figure 5a), presented a smooth surface and possessed macrovoids in the porous cross-sectional microstructure as shown in Figure 5b. In Figure 5c, granular formations on the surface of the membrane can be observed, which were as a direct result of the inclusion of PMAA side groups covalently bonded to PES. The PMAA side groups resulted in increased thermodynamic stability, hence slowing down the liquid–liquid exchange kinetics. This in turn mitigated the formation of macrovoids and resulted in a spongier sublayer, as can be observed in Figure 5d [27]. Upon inclusion of Pd@Fe@HPEI nanoparticles in membrane A1, shown in Figure 5e, these granular formations as observed in A0, were smoothened as chelation with the metal nanoparticles took place. The A1 membrane presented a porous sublayer as a result of increased solvent exchange rates between water and NMP during membrane formation [28]. The inclusion of the Pd@FeAg@HPEI nanoparticles in membrane A2, in Figure 5g, also imparted morphological changes as compared to the previous membranes, because the surface was observed to be rougher. This can be attributed to the enhanced upward movement of the hydrophilic segments (Ag and PMAA) to the surface of the membrane during phase inversion. This process limits the thermodynamics of phase inversion as the latter segments diffuse slowly towards the membrane surface. Consequently, a spongy cross-sectional microstructure of this membrane was formed, as shown in Figure 5h. Other researchers have observed similar membrane formation character and morphology in Ag nanoparticle-containing casting solutions [29]. XPS data of (a) the Fe species, (b) Ag, and (c) Pd (presented in Supplementary Information Figure S2) showed similar binding energies for membrane A2. The oxidation of the nanoparticles can be attributed to their chemical interactions with the carboxylic groups in PMAA-PES. This observation is in agreement with the ATR-FTIR results, where a shift in the carboxylic peaks occurred in the presence of the nanoparticles. Because the penetration by X-rays from the XPS source into the membrane is limited to a few nanometers, it can be concluded that the chelated nanoparticles moved towards the membrane surface with PMAA during phase inversion [9,29,30]. This would be a further advantage as compared to the use of relatively hydrophobic pristine PES.

**Figure 6:**
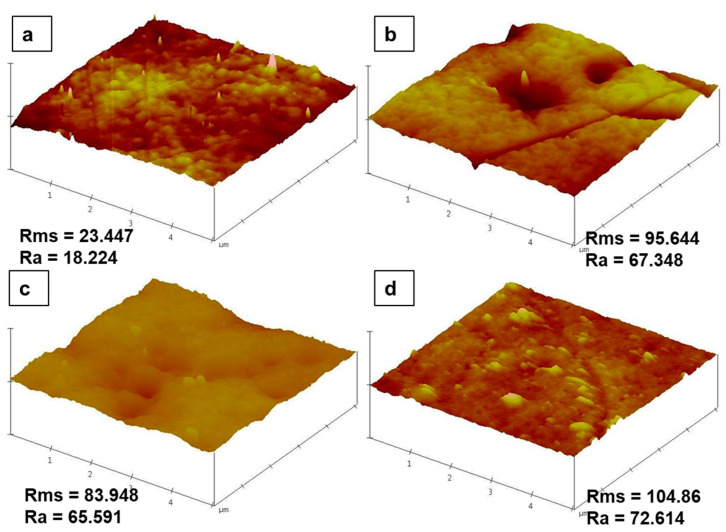
AFM surface topology and roughness for the membranes prepared in this study: (**a**) PES0, (**b**) A0, (**c**) A1, and (**d**) A2. The surface roughness parameters R_ms_ and R_a_ refer to the root square and area mean roughness measurements in nm, respectively.

Figure 6, illustrates the AFM topological morphology and the effect of modification on membrane surface roughness. The pristine PES membrane gave a roughness parameter Ra value of 18.224 nm (Figure 6a). An increased Ra was observed for the A0 membrane, presented in Figure 6b; this was attributed to the PMAA grafts on PES, which were observed in XPS and SEM analyses to exist on the membrane surface. Upon addition of the stabilized Pd@Fe, in membrane A1 (presented in Figure 6c), there was a slight decrease in surface roughness which was attributed to the dispersion of the nanoparticles by PMAA within the membrane matrix. Nonetheless, membrane A2 (Figure 6d), which contained the stabilized Pd@FeAg, presented the highest Ra value; this was attributed to the increased tendency of the solvent-compatible/hydrophilic (PMAA and Ag nanoparticle) segments to diffuse more of this complex to the membrane surface, increasing membrane surface roughness. As such, these AFM results are in agreement with the SEM observations in terms of surface morphology.

### 3.3. Determination of Membrane-Embedded Metal Loading

The amounts of Fe, Ag and Pd in the membranes were quantified as listed in Table 2. For A1, a total metal content of 20.6 mg was measured, and of this, 18.5 mg was observed to comprise Fe. During the synthesis, the aimed metal ratio was 10:1 (Fe:Pd), and the experimental ratio of 9:1 was obtained. Similar amounts were observed for A2, with the total metal adding up to 21.7 mg and the ratios between Fe and the other metals being 10:1 and 8:1 with regard to Pd and Ag, where in both cases a 10:1 ratio was aimed for [3,7].

### 3.4. Membrane Hydrophilicity and Water Interactions

The membranes were subsequently investigated for hydrophilic/hydrophobic character as per contact angle results presented in Table 3. To properly examine the enhancement of hydrophilicity as imparted by the modification of the membranes, these were compared to pristine PES. The pristine PES membrane was observed to have a contact angle of 72°, while the contact angle for membrane A0 was recorded to be 41°. The observation made was a direct result of the inclusion of the hydrophilic PMAA side chains to PES in the membrane matrix [8]. As was observed by SEM, the surface of membrane A1 became rough due to the presence of PMAA crystallites which migrated to the surface during phase inversion. This may have also affected the contact angle results as more water interaction points were created on the membrane surface. The hydrophilicity was not greatly impacted by the inclusion of both nanoparticle systems, further showing the contribution of the particulates to the contact angle results. The Ag-containing Pd@FeAg@HPEI nanoparticle system reduced the contact angle to 35°. This was as a direct consequence of Ag nanoparticles, which increase water interactions due to the large hydration radius on interfacing with water, in addition to the effects of PMAA on the membrane surface [22,27,28,29,30].

Given the enhanced membrane hydrophilicity as presented above, it was also necessary to determine surface free energy changes due to the presence of both nanoparticles systems. Fundamentally, the water contact angle and Δ*G* dictate membrane interactions with water contaminated by organic compounds. These can also dictate the membrane’s activity towards the removal of environmental insults [31]. Table 3 additionally presents the experimental polar and apolar surface components as measured to calculate Δ*G*. For the PES0 membrane, Δ*G* of 48.53 mJ/m^2^ was recorded. This value was observed to decrease in the presence of PMAA side chains within the membrane matrix. As previously discussed from XPS and SEM analyses, the hydrophilic PMAA segments tend to traverse towards the membrane surface during phase inversion in the coagulation bath (4:1 water/IPA). It was the presence of these hydrophilic segments that resulted in decreased surface tensions and hence surface free energies. This decreasing trend in Δ*G* continued with the addition of the nanoparticle systems, and this particulate material introduced defects on the membrane surface. It has been reported in fundamental works that superficial defects increase the interfacial area that can be covered by a water droplet on the membrane surface during measurements [31,32]. This occurrence would then result in decreased surface tension, reducing contact angles, and thus improving membrane–water interactions. It was these interfacial interactions that further informed the membrane’s applicability in terms of solute rejection, water transport, protein adhesion, fouling, and other important parameters. Other works in the literature have investigated these parameters as affected by membrane–water interactions [33,34,35].

### 3.5. Water Uptake and Porosity

**Figure 7:**
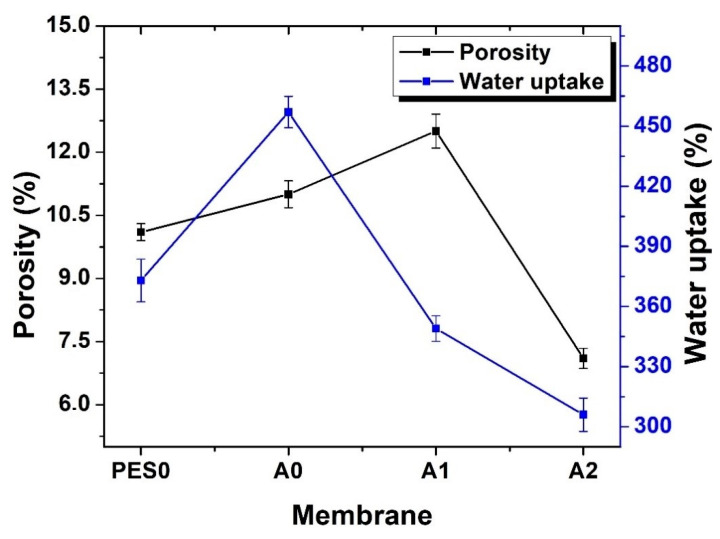
Water uptake and porosity of the fabricated membranes.

Water uptake and porosity are also important properties for a membrane in envisaged water treatment, as they dictate filtration performance and productivity. Figure 7 shows the data computed for water uptake and porosity. The PES0 membrane presented a porosity of 10.1%, and this value increased until A1 due to the respective presence of the PMAA chains and the stabilized Pd@Fe nanoparticles. However, porosity decreased for A2, and this can be attributed to the compact structure it possesses as seen under the SEM analysis. This is in direct contrast to the more porous structure of membrane A1. It is this morphology and porosity, as discussed, that affects water uptake, and these together with the presence of PMAA, increases water adsorption due to the latter’s water-swelling capacity [27]. The increasing water swelling occurred until membrane A1, where it dropped sharply for membrane A2 because of its previously noted compact structure and its low porosity, which reduce its diffusion by water. However, this reduced diffusion may not necessarily be a disadvantage for the intended application, where residence time in the membrane is required to effect degradation of toxic pollutants. This is because these reactions between the nanocomposite and the pollutant molecules have been modeled to occur during diffusion [36].

### 3.6. Pore Size, Pure Water Permeation, and Protein Adhesion

Pore size and pure water permeation are, furthermore, important properties required to assess membrane character. Since these membranes are designed to be reactive and not for size exclusion, reasonable pure water fluxes are required to maximize throughput and minimize the likelihood of fouling during operational life. Furthermore, protein adsorption characteristics of the membranes were studied as high protein interactions may indicate an increased potential for fouling. Table 4 depicts the pore size and pure water fluxes as computed during the fluid saturation and water permeation experiments. The table also presents the amount of BSA adsorbed by the membranes from batch mode experiments. It can be observed that pore size and pure water flux decreased with the modification and inclusion of the nanoparticles. These results can be attributed to the presence of PMAA side chains and nanoparticles which affected the morphology and internal microstructure of the final membranes. As a direct result of this, the porosity decreased, affecting water transport and the recorded water permeate fluxes. The pure water permeate fluxes were observed to decrease due to the modifications which reduce the rates of diffusion of water through the membrane matrix. However, it is also worth noting that membranes A1 and A2 still possessed higher pure water permeate fluxes than PES0. This parameter is related to the enhanced hydrophilicity as possessed by the two membranes [28,36,37].

The BSA molecular adhesion to the membranes is known to affect the formation of biofilms and thus the membrane’s biofouling propensity. This parameter was observed to be the highest for the pristine membrane (10.3 mg cm^−2^). This was due to the relatively hydrophobic nature of PES, which encourages hydrophobic electrostatic interactions between BSA and the pristine membrane. For membrane A0, protein adhesion was observed to be lower compared to the value obtained for PES0. This was attributed to the hydrophilicity imparted by the PMAA side chains on PES. Because the batch mode protein adsorption experiments were carried out at pH 7, this resulted in the deprotonation of both the PMAA chains and BSA molecules. This meant that the PMAA side chains and BSA were both negatively charged, increasing repulsion forces, thus reducing attractions between the membrane and BSA [9]. This trend continued for the membranes containing nanoparticles, as protein adsorption dropped even further to 1.42 and 0.62 µg cm^−2^, as observed for A1 and A2, respectively. The presence of Ag nanoparticles in the A2 membrane was instrumental in further reducing protein adsorption due to their bioactive properties. This observation meant that the Ag nanoparticles-containing membrane possesses the properties required to mitigate the formation of biofilms and possible fouling. Other works in the literature have reported similar results [17,27,29].

## 4. Conclusions

The aim of this work was to develop a new synthesis method for PMAA-PES nanocomposite membranes containing HPEI-stabilized Pd@Fe and Pd@FeAg nanoparticles. The prepared nanoparticles and composites were characterized by FTIR, XPS, and AFM, which indicated the stabilization, presence, and chemical support of the zerovalent nanoparticles within the PMAA-PES membrane matrices. The nanocomposite membranes exhibited enhanced hydrophilicity, pore size, porosity, water uptake, and pure water permeation, while exhibiting reduced protein adhesion. As such, the properties as gained through the modification, are desirable for application of these nanocomposite membranes in water treatment such as for the catalytic removal of toxic dyes, pesticides, herbicides, and other emerging and persistent organic compounds in water.

## Figures and Tables

**Table 1:** Designations for the membranes.

Membrane	
Short Name	Long Name
PES0	Pristine PES
A0 (Ctrl)	PMAA-PES (Control)
A1	Pd@Fe@HPEI/PMAA-PES
A2	Pd@FeAg@HPEI/PMAA-PES

**Table 2:** Amount of metal embedded in the nanocomposite membranes.

Membrane	Fe (mg)	Pd (mg)	Ag (mg)	Total (mg)
A1	18.5 ± 0.7	2.1 ± 0.2	-	20.6 ± 0.9
A2	16.5 ± 0.4	1.7 ± 0.1	2.1 ± 0.2	21.7 ± 0.7

**Table 3:** Contact angle, surface tension, and surface free energy measurements for the membranes before and after the inclusion of the nanoparticles.

Membrane	Contact Angle	Total ΔG (mJ/m^2^)	Apolar	Polar
PES0	72 ± 4	48.53	22.16	26.37
A0	41 ± 2	43.18	21.64	21.54
A1	38 ± 2	41.47	24.59	16.88
A2	35 ± 2	40.79	25.21	15.58

**Table 4:** Pore size, pure water permeation, and protein adsorption data for the fabricated membranes.

Membrane	Pore Size (µm)	Pure Water Flux (L/m^2^ h)	BSA Adsorption (µg cm^−2^)
PES0	0.280	89 ± 6	10.3 ± 0.7
A0	0.377	277 ± 13	3.96 ± 0.5
A1	0.227	136 ± 9	1.42 ± 0.2
A2	0.022	107 ± 5	0.62 ± 0.1

## Supplementary Materials

The following are available online at https://www.mdpi.com/2077-0375/10/9/232/s1, Figure S1: Histograms of size distribution for the nanoparticles: (a) Pd@Fe@PMAA, (b) Pd@Fe@HPEI, and (c) Pd@FeAg@HPEI, Figure S2: Deconvoluted XPS spectra for membrane A2: (a) Fe species (b) Ag, and (c) Pd.
