# Supplementary material for: The Synthesis and Characterization of Novel Bi-/Trimetallic Nanoparticles and Their Nanocomposite Membranes for Envisaged Water Treatment
Source: Membranes (Basel). 2020 Sep 14;10(9):232. doi: 10.3390/membranes10090232 (PMC7559779; doi:10.3390/membranes10090232)
Supplement: Supplementary file 1 [file membranes-10-00232-s001.pdf]

Journal: Membranes (ISSN 2077-0375)

The Synthesis and Characterization of Novel Bi-/Trimetallic Nanoparticles and Their Nanocomposite Membranes for Envisaged Water Treatment

Supplementary data

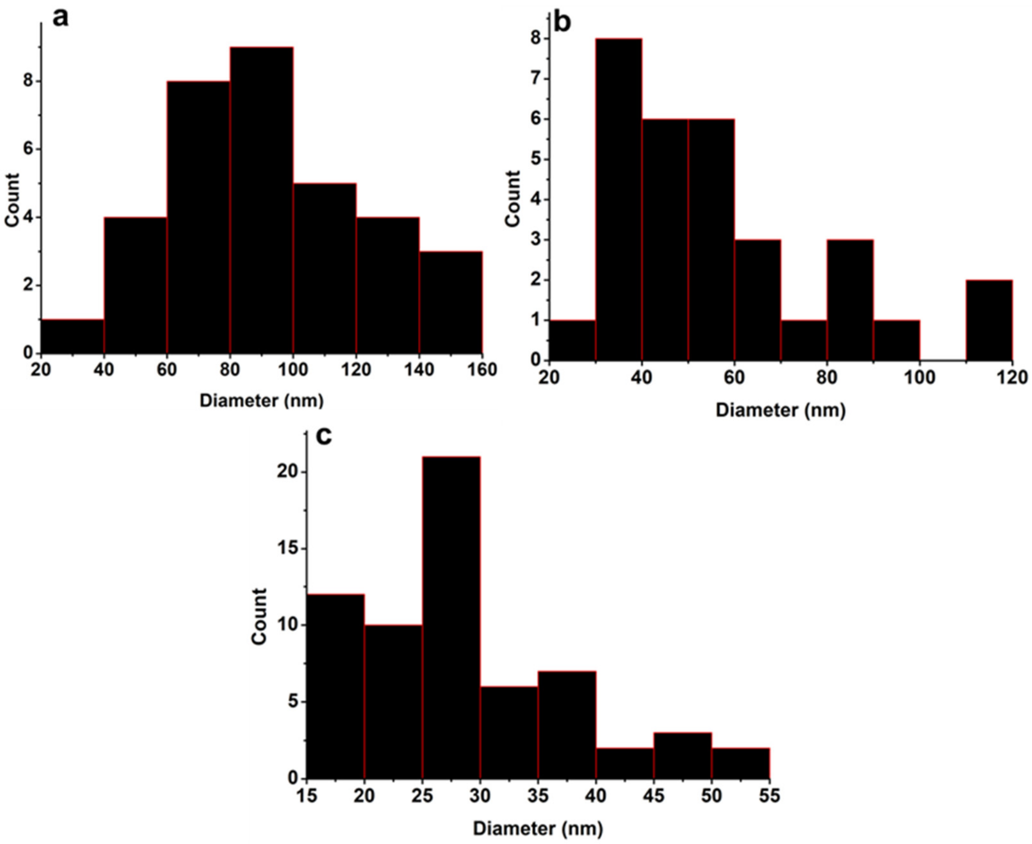

Figure S1. Histograms of size distribution for the nanoparticles: (a) Pd@Fe@PMAA, (b) Pd@Fe@HPEI, and (c) Pd@FeAg@HPEI.

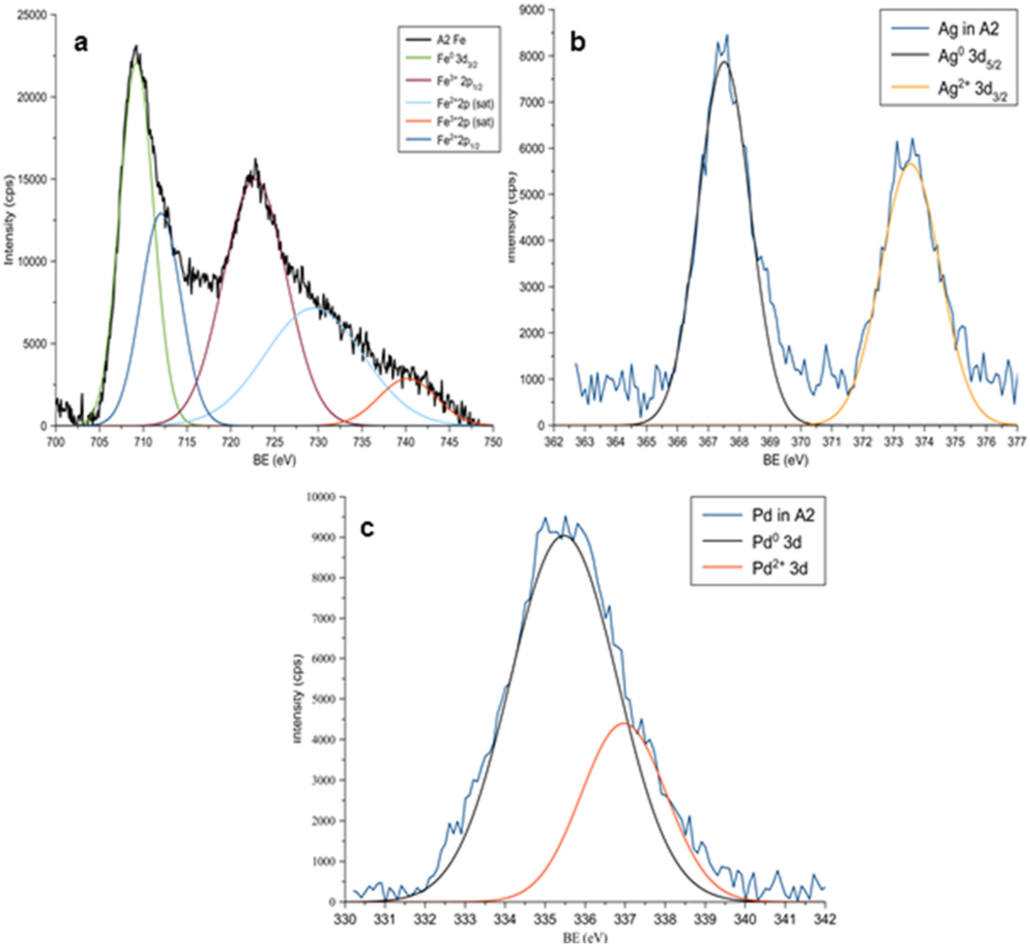

Figure S2. Deconvoluted XPS spectra for membrane A2: (a) Fe species (b) Ag, and (c) Pd.
